# Adequacy of usual macronutrient intake and macronutrient distribution in children and adolescents in Spain: A National Dietary Survey on the Child and Adolescent Population, ENALIA 2013–2014

**DOI:** 10.1007/s00394-018-1676-3

**Published:** 2018-05-22

**Authors:** Ana M. López-Sobaler, Aránzazu Aparicio, Josefa Rubio, Victoria Marcos, Rosa Sanchidrián, Sara Santos, Napoleón Pérez-Farinós, M. Ángeles Dal-Re, Carmen Villar-Villalba, Maria José Yusta-Boyo, Teresa Robledo, José Javier Castrodeza-Sanz, Rosa M. Ortega

**Affiliations:** 1 Spanish Agency for Consumer Affairs, Food Safety and Nutrition, Ministry of Health, Social Services and Equality, Alcalá 56, 28071 Madrid, Spain; 2 Department of Nutrition and Food Science, Faculty of Pharmacy, Complutense University of Madrid, Plaza Ramón y Cajal s/n, 28040 Madrid, Spain; 3 Department of Public Health and Psychiatry, Faculty of Medicine, University of Málaga, Boulevard Louis Pasteur, 32, 28071 Málaga, Spain

**Keywords:** Dietary surveys, Usual intake, Nutrient intake, Europe, Child, Adolescent

## Abstract

**Objectives:**

To describe the nutritional profile and assess the National Dietary Survey on the Child and Adolescent Population project in Spain (ENALIA) regarding usual total energy and macronutrient intake.

**Methods:**

A cross-sectional nationally representative sample of 1862 children and adolescents (age 6 months to 17) was surveyed between 2013 and 2014 following European methodology recommendations. Dietary information was collected using two methods, dietary records (for children from age 6 months to 9 years) and 24-h dietary recall (participants age 10 and older). Usual intake was estimated by correcting for within-person intake variance using the Iowa State University (ISU) method. A probability analysis was used to assess compliance with dietary reference intakes in the target population.

**Results:**

Protein consumption in the age 1–3 group as a percentage of total energy exceeded the upper limit of the Acceptable Macronutrient Distribution Range (AMDR) by 4.7% for boys and 12.1% for girls. 42.9% of girls age 4–8 were under the lower limit of the AMDR for carbohydrates. 43.4% of boys and 46.9% of girls between 4 and 17 exceeded the AMDR in total fat intake, saturated fatty acids (SFAs) accounting for 12.3% of total energy.

**Conclusions:**

The results suggest that Spanish children and adolescents could improve macronutrient distribution by reducing fat and increasing carbohydrate intake across all age groups, and decreasing protein intake, especially in young children.

**Electronic supplementary material:**

The online version of this article (10.1007/s00394-018-1676-3) contains supplementary material, which is available to authorized users.

## Background

Unhealthy diet is one of the most important risk factors associated with non-communicable diseases (NCDs) and is responsible for high morbidity and mortality worldwide [1, 2]. NCD prevention and decreasing risk factors must be addressed in an integral way at all ages but childhood and adolescence are key stages in establishing health habits that will carry into adulthood [3–5].

The upward trend in child obesity and its correlation with cardiovascular health is particularly worrisome [6–9]. Spain, together with other Southern European countries (Greece, Italy and Portugal) is among the European countries with the highest overweight and obesity rate [10, 11]. However, the most recent data collected in Spain between 2011 and 2015 (ALADINO 2015 [12]) indicates a statistically significant decrease in the number of overweight boys and girls between the ages of 6 and 9. Even so, it remains high at 23.2% (22.4% for boys and 23.9% for girls) and the obesity rate remained stable at 18.1% (20.4% for boys, and 15.8% for girls) as per WHO growth standards [13]. When IOFT reference standard [14] is used the prevalence of overweight is similar to the previous, 21.8% (21.5% for boys and 22.2% for girls) but the obesity rate is lower, 11.2% (10.6% for boys and 11.8% for girls) [12]. This trend needs to be confirmed by future study.

Dietary surveys collect information on food intake and eating habits from which estimates of nutrient intake for different population groups are made. However, assessing long-term dietary information (‘usual intake’) is no easy task [15]. Repeated short-term measurements are needed to ensure valid estimates of usual dietary intake for different population groups. Measurements such as 24-h dietary recall or food records proved to be less prone to systematic bias compared with Food Frequency Questionnaires (FFQs) [16]. In addition, some methods have been developed to estimate usual dietary intake based on repeated short-term measurements [17, 18] but few studies, especially focusing on children, have applied them.

In Spain, several dietary surveys conducted at regional level focus on children and adolescents, although the adjustments needed to obtain usual intake estimates were not always applied [19, 20]. The most recent reference survey of this population group at national level was the ENKID study conducted between 1998 and 2000 [20] which estimated usual dietary intake. The results showed a macronutrient distribution with high fat and low carbohydrate intake as well as shifts in eating habits moving away from the Mediterranean Diet. Globalization and ensuing cultural changes and the latters’ influence on food and lifestyle over the past 20 years make it necessary to update this information.

Specifically, inadequate dietary nutrient intake (such as high protein or fat, or low fibre intake) has been linked to a greater risk of obesity in children and adolescents [21–24] and the evidence is even stronger in the adult population [25–29]. Thus, not only the quantity of macronutrient intake is relevant for health but the quality of the diet is important as well [6, 30, 31] such as fat profile, carbohydrates (fibre and simple and complex carbohydrates) and protein (animal and plant). Spain’s Estudio Nacional de Alimentación en Población Infantil y Adolescente (National Dietary Survey on the Child and Adolescent Population—ENALIA) was designed to estimate the usual intake of energy and nutrients and to gain insight into the dietary habits of this target population. The project forms part of the “EU Menu Project” [32], a European project coordinated by the European Food Safety Agency (EFSA) and was conducted in accordance with the agreed European methodology guidance.

The core objective of this study is to provide recent data on the usual energy and macronutrient intake and macronutrient distribution in the diet of Spanish children and adolescents age 6 months to 17 years, evaluated based on compliance with international requirements. An overview of the usual micronutrient intake of the same segment of Spanish children and adolescents has been described elsewhere [33]. Together, these studies will provide the national reference to guide future nutritional interventions targeting this segment of the Spanish population.

## Methods

### Study design

The ENALIA study is a cross-sectional survey conducted in Spain on a nationally-representative sample of children and adolescents designed to collect food consumption data and information on eating habits. The ENALIA study was performed under the umbrella of the Agencia Española de Consumo, Seguridad Alimentaria y Nutrición—AECOSAN (Spanish Agency for Consumer Affairs, Food Safety and Nutrition) between November 2012 and July 2014. It was conducted in line with EFSA’s “EU Menu Project” [32] guidance recommendations and two previous European documents on collection and assessment of food consumption data [16, 34]. The study design and survey protocol are reported elsewhere [33, 35]. The main features are summarised below.

The study was conducted in accordance with the guidelines laid down in the Helsinki Declaration. Depending on the age of participants, information was given to parents, tutors, or other legal representatives, and all participants gave their consent before proceeding with the interviews. This study was approved by the Spanish Agency for Consumer Affairs, Food Safety and Nutrition (AECOSAN), an agency attached to the Spanish Ministry of Health, Social Services and Equality. In accordance with the Spanish Ethical Review System, ethical approval was not needed since this is a population-based survey not requiring any intervention or the taking of human biological samples. All data were anonymous.

### Samples

**Fig. 1:**
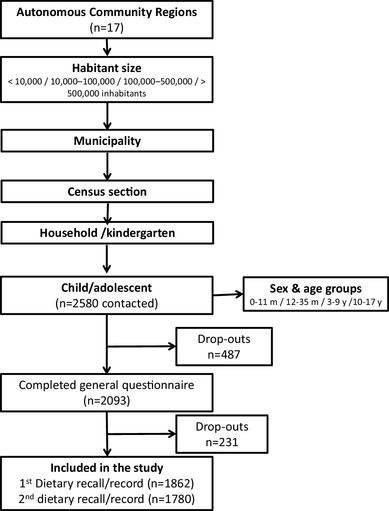
Stratified multistage cluster-sampling in ENALIA study, 2013–2014. Brief description of response rate: a total of 2580 households were contacted and 2093 of them completed the general questionnaire. This corresponds to an initial response rate of 81.1%. 1862 of the households that completed the general questionnaire (72.2% of the total households contacted) participated in the first dietary record/24-h recall. The second reminder was filled out by 1780 individuals, 69.0% of the children contacted

Sampling was population-based and representative of the under 18 Spanish population. The target population was comprised of people between 6 months and 17 years of age living in private households. According to EU Menu guidance recommendations [16, 32], the target sample size had to include a minimum of 260 subjects (130 males and 130 females) in each group of study (infants, toddlers, other children and adolescents). This figure was established taking into account the EFCOSUM project [36] and Kroes et al. [37] recommendations about the accuracy of estimates of high and low consumption levels. Since the inclusion of a larger number of subjects is strongly recommended, in particular for the most populated EU Member States [16], ENALIA was designed to achieve the double of the recommended sample size in other children and adolescents groups [35].The sample was selected using a multi-stage cluster sampling design, municipality of residence being the first stage followed by census section and household/kindergarten (only in the case of children in day-care). All sample units for these clusters were randomly selected. Population censuses from January 2013 collected by the National Statistics Institute of Spain were the source of information for the census sections. The sample was first stratified by geopolitical region and then by town size (< 10,000, 10,000–100,000, 100,000–500,000, and over 500,000 inhabitants). The last sample unit (participants) was stratified by gender and age (50% boys, 50% girls; 0–11 months, 12–35 months, 3–9 years, and 10–17 years). Efforts were made to achieve the sample size while minimizing the non-response rate. The recruitment process consisted of five contacts: an initial contact with households by phone or at nurseries/kindergartens (for children under 3 years of age), a second contact by letter (with details about the study and consent to participate, including the general questionnaire and the diary/24-h recall), two further contacts by phone (the third to confirm consent and administer the general questionnaire and the fourth for the first diary record/24-h recall) and a fifth and final in-person contact at the household (where the second diary record/24-h recall and anthropometric study were conducted). The general questionnaire administered to the entire potential sample, regardless of willingness to participate, enabled a comparison of the characteristics of those who did not answer the first diary record/24-h recall with those who did answer (Fig. 1).

To allow for inter-seasonal variability in consumption patterns, subjects were uniformly distributed over the four different seasons, and the schedule was organized in such as way as to include the appropriate proportion of weekdays and week-end days at the population group level. The sample was also distributed uniformly over the weeks in each month.

### Dietary intake assessment

Dietary information per se was collected between Spring 2013 and Spring 2014. Special techniques were used to accommodate a detailed description and quantification of foods. Dietary assessment was based on two non-consecutive 1-day food diaries for children age 6 months to 10 years, and two 24-h dietary recalls for 11 to 17-year-old children and adolescents, separated by at least 14 days to ensure that information best resembled usual dietary intake. In the former, the child’s parents or caregivers were the respondents and, in the latter, adolescents were the respondents with parents and caregivers present during interviews. A food propensity questionnaire (FPQ) especially designed for infants and adolescents was used to supplement both methods [32]. The FPQ included information about specific food/beverages that stood out for their nutritional value or potential risk, specific food groups such as fruits and vegetables and questions on the intake of dietary supplements. It included 44 questions about food groups and selected food items (e.g. chocolate, processed baked goods) and 13 questions related to food supplements [35]. Interviews were conducted using specific software called ENIA-soft (version 5.0, Demométrica SL, Madrid, Spain) in computer-assisted interviews by trained interviewers and nutritionists/dieticians. ENIA-soft was previously used in other Spanish food surveys [38] and was adapted to meet the study objectives. This new version was validated during the pilot phase of the project. Information from standard questions already incorporated into the software tool was collected for descriptions such as cooking and processing method (boiling, baking, canning, smoking, etc.), qualitative information (light, lactose free, gluten free, etc.), fortification agents (vitamins, chemical substances, special fatty acids, etc.), and preparation-production place (industrial, prepared at a café, bakery, etc.).

Amounts consumed were estimated using a Spanish picture book, supplemented with measurement in grams, common household measurements and portions indicated in standard recipes. The Spanish picture book included pictures of portion sizes and dishes of 57 different food products or simple recipes and was validated (perception) by two sets of volunteers in two sessions: parents and adolescents [35]. A database was built that contained the weight of each of the portions illustrated in the book. Depending on the product, weight data of more than 200 different household measurements or commercial units (spoon, glass, pinch, handful, drop, bottle, can, slice, etc.) were also included. Finally, some food weights were available from manufacturer’s information (tins, beverages, pre-packaged foods, etc.). As quantification is a key factor in the accuracy of food consumption data, ENIA-soft has integrated the picture book, standard recipes, and the database with all food weights and household measurements. There were no exclusively breastfed infants but 41.4% of the recalls among children aged 6–12 months and 3.4% of those aged 1–3 years included breast milk. For partially breastfed children, 120 g of breast milk per feeding was computed [39]. 74.7% of the mid-morning snacks and 23.8% of lunches on working days were consumed at school. For children under 10 years, parents and caregivers were responsible for completing the food diaries and the other questionnaires, and requested collaboration and/or information from other proxy persons about the child’s out-of-home diet, such as school canteen personnel. Parents and caregivers were also present during interviews with adolescents to provide details about meals prepared at home.

Nutrient intakes were calculated using the Spanish Food Composition Tables [40] and were supplemented with additional data on nutrient composition for specific brands and enriched/fortified foods. There was no information on quantities or brand supplements used so nutrient intake data only pertained to food. This analysis focuses on proteins, carbohydrates, fibre, total fat, and different types of fatty acids (polyunsaturated fatty acids—PUFAs, mono unsaturated fatty acids—MUFAs and saturated fatty acids—SFAs) and cholesterol in the diet.

### Handling of misreporting

The plausibility of energy intake was assessed using the Goldberg cut-off method [41] updated by Black [42], following the methodology applied in other European studies on children [43]. Basal metabolic rate (BMR) was estimated by means of Schofield equations [44] taking age, sex, body height, and weight into account [38, 39]. Age- and sex-specific cut-offs for children and adolescents were calculated as suggested [45] using specific reference values for the within-subject coefficient of variation (CV) for energy intake (EI) and physical activity (PAL) as given by Black [42], and CV of BMR as given by Nelson et al. [46]. PAL values were adopted following EFSA recommendations [32]. Under-reporters were identified as those with EI/BMR ratios under 0.73–1.08, while over-reporters were identified by EI/BMR ratios above 2.29–2.88 depending on the subject’s age and sex. As per EFSA recommendations [32], we did not exclude potential misreporters from the analysis. Exclusion of misreporters from datasets would introduce bias and were, therefore, identified, but not excluded from the dataset.

### Other information

A general questionnaire addressed socio-demographic data such as participants’ birth date, place/country of origin and that of their parents, academic level and profession of parents and about the health status of the participant (e.g., special diet, drug use and chronic or acute diseases).

Anthropometric data were measured at the interviewee’s home by trained interviewers who followed standardized procedures [38]. A stadiometer was used to measure stature in subjects aged 2 years and older and an infantometer was used to measure the recumbent length of subjects aged 6–24 months. Stature and length were measured in centimetres. A digital weight scale with accuracy of 0.1 kg was used. BMI was calculated and the WHO reference standards [13, 47] was used to calculate BMI-Z scores.

### Statistical analysis

Sample weight factors for each participant were calculated to account for non-responses and to weight the sample to known population demographic characteristics. A *p* value of < 0.05 indicated statistical significance. The Kolmogorov–Smirnov test was used to check the distribution normality of age and anthropometric and EI/BMR data. The Student’s *t* test or the Mann–Whitney *U* test, depending on whether or not data were distributed normally, were used to examine differences between males and females. Categorical variables were compared using the *χ*2 test. Statistical analyses were performed using the statistical software package SPSS version 20.0 for Windows.

The Iowa State University method (ISU) [48] was used to adjust intake data taking both between and within-individual variability in dietary intake into account. It was implemented using the PC-SIDE software (version 1.0, Department of Statistics, Centre for Agricultural and Rural Development, Ames, IA, USA), which was designed for this purpose. This program estimated the percentiles of usual daily nutrient intake distributions as well as the proportion above or below the defined dietary reference intake (DRI) cut-off values [49, 50]. The day of the week, the interview day (day 1 or day 2), season, and sampling weighting factor were taken into account when adjusting dietary data and stratifying by sex and age group.

The distribution of participants’ usual energy and macronutrient dietary intake was presented as means and standard deviations, median and 10th, 25th, 75th, and 90th percentiles by age group and sex. Both absolute amounts (g/day) and amounts relative to total energy intake (%En) of participants were evaluated according to recommended reference values. The estimated average requirement (EAR) cut-off point method was used to assess protein and carbohydrate adequacy [50]. Spanish DRIs do not provide the estimated average requirement (EAR) values we required for our analyses so we needed to choose another set of dietary recommendations. We recently analysed the adequacy of micronutrient intake in this same group of Spanish children using the IOM’s DRIs [33]. We decided to use the same set of recommendations to analyse the same age groups. The IOM’s DRI are regularly updated and frequently used compared to reference values provided by other scientific bodies. However, in cases where EFSA [51] or others (such as ESPGHAN) [52] publish a reference value, the adequacy estimate was made according to that recommendation. Acceptable macronutrient distribution ranges (AMDR) were used to evaluate the distribution of participants relative to the total energy intake percentage (%En) from proteins, carbohydrates, and total fat [50]. The proportion of the population with usual intake under the EAR provides an estimate of the proportion of the group whose intake does not meet the nutrient requirement while the population with usual intake in the AMDR represents the proportion whose intake is associated with higher risk of chronic disease. Finally, the fat profile was estimated and the ratio (PUFAs + MUFAs)/SFA was modelled. To check whether misreporting skews findings, comparisons of nutrient intakes were repeated using only the plausible reporters (supplemental tables 1 and 2).

## Results

The final ENALIA sample included 1,862 children between the ages of 6 months and 17.9 years, 1,780 of whom provided two 1-day food diaries/24-h dietary recalls. The overall response rate was 69.0% (Fig. 1). Sample characteristics by gender are described in Table 1. The percentage of under-reporters ranged from 0.6% (children aged 6–12 months) to 19.8% (adolescents aged 14–17). On the other hand, overestimation was higher in infant children (15.4%) and lower in adolescents (0.4%) (Table 2). The data presented in the rest of this report have not been adjusted for under-reporting.

**Table 1:** Characteristics of the studied sample in the ENALIA Study, 2013–2014 (National Dietary Survey on the Child and Adolescent Population)

	Total	Boys	Girls
*n*	1862	967	895
Age (years), *X* ± SD	8.8 ± 4.9	8.9 ± 4.9	8.8 ± 4.8
Age group, *n* (%)			
6–12 months	292 (15.7)	138 (14.3)	154 (17.2)
1–3 years	407 (21.9)	218 (22.5)	189 (21.1)
4–8 years	418 (22.5)	211 (21.8)	207 (23.1)
9–13 years	470 (25.2)	243 (25.1)	227 (25.4)
14–17 years	275 (14.8)	157 (16.2)	118 (13.2)
Anthropometric characteristics			
Weight (kg), X ± SD	34.25 ± 18.15	35.36 ± 19.23	33.07 ± 16.88*
Height (cm), X ± SD	131.6 ± 30.4	133.0 ± 31.5	130.1 ± 29.1*
BMI (kg/m^2^), X ± SD	18.1 ± 3.1	18.1 ± 3.0	18.0 ± 3.1
Z-BMI, X ± SD	0.33 ± 1.20	0.37 ± 1.27	0.29 ± 1.13
Community size^a^*n*, (%)			
< 10,000	358 (19.2)	184 (19.0)	174 (19.4)
10,000–100,000	761 (40.9)	380 (39.3)	381 (42.6)
100,000–500,000	466 (25.0)	256 (26.5)	210 (23.5)
> 500,000	277 (14.9)	147 (15.2)	130 (14.5)
Father’s highest educational level *n*, (%)			
Mandatory or less^b^	573 (31.1)	300 (31.4)	273 (30.8)
Secondary	536 (29.1)	283 (29.7)	253 (28.6)
University	731 (39.7)	371 (38.9)	360 (40.6)
Mother’s highest educational level *n*, (%)			
Mandatory or less^b^	455 (24.5)	239 (24.8)	216 (24.2)
Secondary	504 (27.1)	256 (26.6)	248 (27.8)
University	898 (48.4)	469 (48.7)	429 (48.0)

*ENALIA* Encuesta Nacional de ALimentación en Población Infantil y Adolescente de España (National Dietary Survey in Spanish Children and Adolescents), *SD* standard deviation, *BMI* body mass index, *Z-BMI z* score for BMI-for-age

^a^Number of inhabitants

^b^≤10 years of education

*p < 0.05, significant differences between sex groups

**Table 2:** Data related to misreporting energy intake in the ENALIA Study, 2013–2014 (National Dietary Survey on the Child and Adolescent Population)

	Total	Boys	Girls
EI/BMR *X* ± SD			
6–12 months	2.22 ± 0.47	2.22 ± 0.43	2.21 ± 0.51
1–3 years	1.88 ± 0.50	1.87 ± 0.46	1.90 ± 0.55
4–8 years	1.62 ± 0.51	1.64 ± 0.50	1.59 ± 0.52
9–13 years	1.43 ± 0.46	1.41 ± 0.46	1.45 ± 0.45
14–17 years	1.30 ± 0.38	1.32 ± 0.38	1.27 ± 0.37
Underreporters *n* (%)			
6–12 months	2 (0.6)	0 (0.0)	2 (1.3)
1–3 years	11 (2.6)	3 (1.6)	8 (3.7)
4–8 years	29 (6.9)	13 (6.2)	16 (7.7)
9–13 years	52 (11.0)	29 (12.0)	23 (10.1)
14–17 years	55 (19.8)	37 (23.6)	18 (15.3)
Plausible reporters, *n* (%)			
6–12 months	243 (84.0)	129 (93.5)	114 (74.0)*
1–3 years	380 (93.5)	211 (96.8)	169 (89.8)*
4–8 years	381 (91.1)	197 (93.4)	184 (88.9)
9–13 years	408 (86.8)	211 (86.8)	197 (86.8)
14–17 years	219 (79.8)	119 (75.8)	100 (84.7)*
Overreporters, *n* (%)			
6–12 months	47 (15.4)	9 (6.5)	38 (24.7)*
1–3 years	16 (3.9)	4 (1.6)	12 (6.5)*
4–8 years	8 (1.9)	1 (0.5)	7 (3.4)*
9–13 years	10 (2.2)	3 (1.2)	7 (3.1)
14–17 years	1 (0.4)	1 (0.6)	0 (0.0)

*ENALIA* Encuesta Nacional de ALimentación en Población Infantil y Adolescente de España (National Dietary Survey in Spanish Children and Adolescents), *SD* standard deviation, *BMI* body mass index, *Z-BMI z* score for BMI-for-age, *EI/BMR* observed energy intake/basal metabolic rate ratio

*p < 0.05, significant differences between sex groups

Usual intake (of food and beverage only) of energy and macronutrients and inadequate intake by age group and sex is presented in Table 3. Only 0.1% of girls in the 14–17 year old bracket did not meet the EAR for proteins, and 0.2% of girls in the 1–3 year old bracket did not meet the EAR for carbohydrates. As the modelled PUFA + MUFA/SFA ratio indicates, the median (P10th–P90th) usual score in the diet of Spanish children and adolescents was similar across gender and all age groups, except for the 6–12 month group where the highest score was exhibited [2.2 (1.4–5.4) for boys and 2.3 (1.4–4.3) for girls] (Table 4).

**Table 3:** Usual intakes (from food and beverage sources only) of energy and macronutrients in Spanish children and adolescents and inadequate intakes

	Boys								Girls							
	mean ± SD	P10	P25	P50	P75	P90	EAR	<EAR	mean ± SD	P10	P25	P50	P75	P90	EAR	< EAR
Energy (kcal)																
6–12 months	1109 ± 161	906	998	1104	1214	1317			1019 ± 138	842	926	1020	1113	1196		
1–3 years	1479 ± 214	1209	1332	1473	1620	1756			1380 ± 173	1164	1259	1372	1491	1606		
4–8 years	1847 ± 211	1583	1701	1839	1984	2121			1652 ± 157	1456	1543	1646	1754	1856		
9–13 years	2109 ± 229	1819	1952	2104	2261	2406			1879 ± 225	1594	1724	1873	2027	2170		
14–17 years	2375 ± 406	1866	2092	2359	2639	2904			1881 ± 295	1506	1678	1875	2077	2263		
Protein (g)																
6–12 months	33.9 ± 9.5	22.4	27.1	33.0	39.7	46.5			31.3 ± 7.6	21.9	26.0	30.9	36.2	41.4		
1–3 years	61.6 ± 10.5	48.6	54.2	61	68.2	75.3	11	0.0	59.3 ± 11.7	45.2	51	58.3	66.5	74.7	11	0.0
4–8 years	78.6 ± 11.7	64.0	70.4	78.0	86.1	94.0	19	0.0	70.3 ± 7.5	61.0	65.1	69.9	75.1	80.1	19	0.0
9–13 years	88.7 ± 10.1	76.1	81.7	88.2	95.2	101.9	34	0.0	78.7 ± 11.2	64.6	71.0	78.4	86.1	93.3	34	0.0
14–17 years	104.6 ± 15.6	85.2	93.7	103.8	114.6	125.0	52	0.0	82.6 ± 13.6	65.6	73.2	82.1	91.4	100.3	46	0.1
Protein (g/kg)																
6–12 months	4.66 ± 0.97	3.61	4.05	4.53	5.28	6.14			4.79 ± 1.04	3.64	4.08	4.72	5.36	6.25		
1–3 years	3.25 ± 0.82	2.23	2.72	3.16	3.66	4.40			3.01 ± 0.76	2.01	2.36	3.06	3.53	4.03		
4–8 years	2.21 ± 0.57	1.52	1.76	2.13	2.62	3.02			1.98 ± 0.55	1.34	1.61	1.92	2.30	2.69		
9–13 years	1.72 ± 0.37	1.24	1.45	1.71	1.93	2.21			1.52 ± 0.33	1.11	1.28	1.50	1.73	1.96		
14–17 years	3.65 ± 0.96	2.57	3.09	3.58	4.24	4.94			3.56 ± 0.84	2.55	3.03	3.52	4.10	4.68		
Carbohydrates (g)																
6–12 months	147 ± 22	119	132	146	161	176			133 ± 23	104	118	133	148	162		
1–3 years	173 ± 26	140	154	171	189	207	100	0.0	161 ± 22	133	145	160	176	190	100	0.2
4–8 years	214 ± 32	174	191	212	234	255	100	0.0	188 ± 26	155	169	186	205	223	100	0.0
9–13 years	246 ± 31	206	224	245	266	286	100	0.0	219 ± 29	183	199	217	237	256	100	0.0
14–17 years	276 ± 55	208	238	273	311	348	100	0.0	217 ± 34	174	193	215	239	261	100	0.0
Fiber (g)																
6–12 months	9.2 ± 3.4	4.9	6.8	9.1	11.5	13.7			8.5 ± 2.8	5.3	6.6	8.2	10.0	12.1		
1–3 years	12.6 ± 3.6	8.5	10.1	12.2	14.7	17.4			11.8 ± 3.8	7.4	9.1	11.3	14	16.9		
4–8 years	15.7 ± 2.7	12.4	13.7	15.4	17.3	19.2			15.3 ± 3.1	11.5	13.0	14.9	17.1	19.4		
9–13 years	18.4 ± 3.8	13.8	15.7	18.0	20.7	23.3			17.7 ± 4.7	12.1	14.3	17.2	20.5	24.0		
14–17 years	21.3 ± 5.9	14.4	17.1	20.6	24.7	29.0			18.4 ± 5.1	12.3	14.7	17.8	21.4	25.1		
Fats (g)																
6–12 months	40.8 ± 8.1	30.9	35.1	40.2	45.9	51.5			38.6 ± 5.7	31.6	34.7	38.3	42.3	46.1		
1–3 years	57.7 ± 11.9	42.8	49.3	57.1	65.4	73.3			52.8 ± 8.7	42.2	46.7	52.2	58.3	64.3		
4–8 years	71.9 ± 8.7	61.0	65.8	71.5	77.5	83.2			65.4 ± 7.2	56.4	60.4	65.1	70.1	74.8		
9–13 years	81.9 ± 12.8	65.9	72.9	81.3	90.2	98.7			72.6 ± 12.6	57.1	63.8	71.9	80.7	89.2		
14–17 years	89.8 ± 17.5	68.3	77.5	88.7	101.0	112.9			71.9 ± 15.1	52.6	61.3	71.6	82.2	91.7		
SFA (g)																
6–12 months	9.6 ± 4.9	4.4	6.1	8.7	12.2	16.3			9.0 ± 5.0	3.4	5.4	8.2	11.8	15.7		
1–3 years	19.8 ± 7.0	10.9	14.7	19.5	24.6	29.2			18.3 ± 6.5	10.3	13.6	17.7	22.4	26.9		
4–8 years	27.1 ± 4.6	21.4	23.9	26.8	30.0	33.1			24.0 ± 4.0	19.1	21.2	23.7	26.5	29.2		
9–13 years	29.4 ± 5.6	22.5	25.4	29.0	32.9	36.7			26.7 ± 5.4	20.1	22.9	26.3	30.1	33.7		
14–17 years	32.0 ± 7.1	23.2	26.9	31.5	36.5	41.4			24.1 ± 5.1	17.8	20.5	23.8	27.4	30.9		
MUFA (g)																
6–12 months	11.8 ± 4.6	6.3	8.5	11.3	14.6	17.9			11.3 ± 4.0	6.4	8.4	10.9	13.8	16.6		
1–3 years	19.2 ± 6.1	11.6	14.9	18.8	23.1	27.3			17.8 ± 5.0	11.7	14.3	17.5	20.9	24.3		
4–8 years	27.4 ± 4.3	22.1	24.4	27.1	30.1	33.0			25.9 ± 2.6	22.6	24.1	25.8	27.6	29.3		
9–13 years	32.5 ± 5.5	25.7	28.7	32.2	36.0	39.6			28.0 ± 5.1	21.6	24.3	27.6	31.2	34.7		
14–17 years	35.3 ± 7.5	26.2	30.0	34.7	40.0	45.2			29.1 ± 7.0	20.3	24.2	28.9	33.7	38.3		
PUFA (g)																
6–12 months	6.0 ± 1.1	4.8	5.3	5.9	6.7	7.4			5.8 ± 0.9	4.8	5.2	5.7	6.3	7.0		
1–3 years	8.4 ± 2.2	5.9	6.8	8.1	9.6	11.3			7.7 ± 1.3	6.2	6.8	7.5	8.4	9.3		
4–8 years	10.5 ± 0.8	9.5	10.0	10.5	11.0	11.5			9.1 ± 1.7	7.1	7.9	8.9	10.1	11.3		
9–13 years	12.2 ± 2.6	9.1	10.4	12.0	13.8	15.6			10.9 ± 2.3	8.2	9.3	10.7	12.4	14.0		
14–17 years	13.6 ± 2.9	10.1	11.5	13.3	15.3	17.4			11.4 ± 2.5	8.4	9.7	11.2	12.9	14.7		
Cholesterol (mg)																
6–12 months	122 ± 68	46	71	110	163	219			113 ± 67	38	64	101	150	203		
1–3 years	229 ± 88	126	165	218	281	347			220 ± 93	110	151	208	276	347		
4–8 years	328 ± 55	261	290	324	363	400			296 ± 41	245	267	294	322	349		
9–13 years	365 ± 54	298	327	362	400	437			294 ± 79	199	238	287	342	398		
14–17 years	401 ± 73	312	350	397	447	497			331 ± 69	248	282	324	372	421		

*Mean* mean values, *SD* standard deviation, *P* percentile, *PUFA* poly-unsaturated fatty acids, *MUFA* mono-unsaturated fatty acids, *SFA* saturated fatty acids, *EAR* Estimated Average Requirements (IOM, 2005)

**Table 4:** Distribution of relative usual intakes (from food and beverage sources only) of macronutrients (percent of the total energy intake, %En) and PUFA + MUFA/SFA ratio in Spanish children and adolescents and inadequate intakes

	Boys									Girls								
	mean ± SD	P10	P25	P50	P75	P90	AMDR	< AMDR	> AMDR	mean ± SD	P10	P25	P50	P75	P90	AMDR	< AMDR	> AMDR
Protein (%En)																		
6–12 months	12.0 ± 2.0	9.6	10.6	11.9	13.3	14.7				12.1 ± 1.8	9.9	10.8	12.0	13.3	14.5			
1–3 years	16.7 ± 1.9	14.3	15.3	16.5	17.8	19.1	5–20	0.0	4.7	17.2 ± 2.4	14.2	15.5	17	18.7	20.3	5–20	0.0	12.1
4–8 years	17.1 ± 1.5	15.3	16.1	17.1	18.1	19.1	10–30	0.0	0.0	17.1 ± 1.4	15.4	16.1	17.0	18.0	19.0	10–30	0.0	0.0
9–13 years	17.0 ± 1.6	15.1	15.9	16.9	18.0	19.1	10–30	0.0	0.0	16.9 ± 1.9	14.4	15.5	16.8	18.1	19.4	10–30	0.0	0.0
14–17 years	17.8 ± 1.5	16.0	16.8	17.8	18.8	19.8	10–30	0.0	0.0	17.8 ± 1.8	15.6	16.6	17.8	19.0	20.2	10–30	0.0	0.0
Carbohydrates (%En)																		
6–12 months	53.1 ± 4.3	47.4	50.3	53.3	56.1	58.4				52.2 ± 4.2	46.9	49.4	52.2	55.1	57.6			
1–3 years	47.2 ± 4.3	41.7	44.3	47.2	50.1	52.8	45–65	30.7	0.0	46.6 ± 4.4	41	43.7	46.6	49.6	52.2	45–65	35.6	0.0
4–8 years	46.4 ± 3.7	41.6	43.9	46.4	48.9	51.1	45–65	35.7	0.0	45.5 ± 2.9	41.8	43.6	45.5	47.4	49.2	45–65	42.9	0.0
9–13 years	46.6 ± 2.8	42.9	44.7	46.6	48.5	50.2	45–65	28.5	0.0	46.7 ± 3.2	42.6	44.6	46.7	48.9	50.8	45–65	29.7	0.0
14–17 years	46.5 ± 3.2	42.4	44.4	46.6	48.7	50.6	45–65	30.9	0.0	46.3 ± 3.3	42.1	44.1	46.3	48.6	50.6	45–65	34.9	0.0
Fats (%En)																		
6–12 months	33.4 ± 5.1	27.1	29.8	33.1	36.6	40.0				34.2 ± 4.3	28.8	31.3	34.2	37.1	39.7			
1–3 years	34.6 ± 3.4	30.3	32.3	34.6	36.8	38.8	30–40	8.8	5.0	34.6 ± 3.1	30.6	32.5	34.6	36.7	38.6	30–40	7.1	4.4
4–8 years	34.8 ± 2.9	31.2	32.9	34.8	36.8	38.5	25–35	0.0	47.5	35.6 ± 2.8	32.0	33.7	35.6	37.5	39.2	25–35	0.0	58.4
9–13 years	34.7 ± 3.1	30.7	32.6	34.7	36.8	38.7	25–35	0.0	46.3	34.6 ± 2.9	30.9	32.6	34.5	36.5	38.2	25–35	0.0	43.7
14–17 years	33.9 ± 3.4	29.5	31.5	33.8	36.1	38.2	25–35	0.1	36.5	34.0 ± 3.7	29.3	31.5	33.9	36.4	38.6	25–35	0.7	38.6
SFA (%En)																		
6–12 months	8.0 ± 4.2	3.8	5.0	7.0	10.2	14.1				8.2 ± 4.9	2.9	4.5	7.2	11	15.3			
1–3 years	11.9 ± 3.2	7.5	9.6	12.0	14.2	16.0				11.9 ± 3.6	7.2	9.4	12	14.5	16.7			
4–8 years	13.1 ± 1.6	11.1	12.1	13.1	14.2	15.1				13.1 ± 1.9	10.8	11.9	13.1	14.4	15.5			
9–13 years	12.4 ± 1.5	10.5	11.4	12.4	13.4	14.4				12.7 ± 1.6	10.8	11.6	12.7	13.7	14.7			
14–17 years	12.0 ± 1.5	10.1	11.0	12.0	13.0	14.0				11.4 ± 1.2	10.0	10.6	11.4	12.2	12.9			
MUFA (%En)																		
6–12 months	9.7 ± 3.6	5.2	7.1	9.4	11.9	14.5				9.9 ± 3.9	4.9	7	9.7	12.6	15.2			
1–3 years	11.3 ± 2.7	7.8	9.4	11.3	13.1	14.8				11.6 ± 2.5	8.4	9.9	11.5	13.3	14.8			
4–8 years	13.2 ± 1.8	11.0	12.0	13.2	14.4	15.6				14.1 ± 1.5	12.2	13.0	14.0	15.1	16.0			
9–13 years	13.8 ± 1.7	11.6	12.6	13.7	14.9	16.0				13.3 ± 1.3	11.7	12.4	13.3	14.1	14.9			
14–17 years	13.4 ± 1.9	11.0	12.0	13.3	14.6	15.9				13.6 ± 2.1	10.9	12.2	13.6	15.0	16.3			
PUFA (%En)																		
6–12 months	4.9 ± 0.5	4.3	4.6	4.8	5.2	5.5				5.1 ± 0.4	4.6	4.8	5	5.3	5.6			
1–3 years	4.9 ± 0.9	3.9	4.3	4.8	5.5	6.1				5.0 ± 0.7	4.1	4.5	4.9	5.4	5.9			
4–8 years	5.1 ± 0.2	4.9	5.0	5.1	5.2	5.3				4.9 ± 0.7	4.0	4.4	4.8	5.4	5.9			
9–13 years	5.2 ± 0.7	4.3	4.7	5.1	5.6	6.1				5.2 ± 0.9	4.1	4.5	5.1	5.8	6.4			
14–17 years	5.1 ± 0.5	4.4	4.7	5.1	5.4	5.8				5.5 ± 0.9	4.4	4.9	5.4	6.0	6.6			
PUFA + MUFA/SFA																		
6–12 months	3.5 ± 6.02	1.4	1.7	2.2	3.2	5.4				2.7 ± 2.06	1.4	1.7	2.3	3.1	4.3			
1–3 years	1.6 ± 0.45	1.1	1.3	1.5	1.8	2.2				1.6 ± 0.51	1.1	1.2	1.5	1.9	2.3			
4–8 years	1.5 ± 0.21	1.2	1.3	1.4	1.6	1.7				1.5 ± 0.27	1.2	1.3	1.5	1.7	1.9			
9–13 years	1.6 ± 0.25	1.3	1.4	1.6	1.7	1.9				1.5 ± 0.17	1.3	1.4	1.5	1.6	1.8			
14–17 years	1.6 ± 0.21	1.3	1.4	1.6	1.7	1.9				1.8 ± 0.2	1.5	1.6	1.7	1.9	2.0			

*Mean* Mean values, *SD* standard deviation, *P* percentile, *AMDR* acceptable macronutrient distribution ranges (IoM, 2005), *< AMDR* percentage of the participants whose macronutrients values are below the lower range limit, *> AMDR* percentage of the participants whose macronutrients values are above the upper range limit, *PUFA* poly-unsaturated fatty acids, *MUFA* mono-unsaturated fatty acids, *SFA* saturated fatty acids

The distribution of relative usual intake of macronutrients the percent of total energy intake (%En) and the proportion of the population that falls below and above the AMDR by age group and sex are presented in Table 4. Median values for protein were 16.8% (P10th–P90th) (13.9–20.1%) of the total energy intake. EFSA has not established an AMDR for children aged 6–12 months, but ESPGHAN sets 15%En as an upper limit [52]. Considering this latter limit, 7.9% of boys and 6.4% of girls were above the upper limit for protein. For boys, 4.7%, and for girls age 1–3 years, 12.1% were above the specific AMDR for protein. Median values for Carbohydrates were 46.8% (P10th–P90th) (41.2–52.3%) of total energy. The usual median proportion of energy intake from carbohydrates was higher for infants from 6 to 12 months than for the other age groups. The proportion of participants with usual En% intake from carbohydrates below the lower limit of the AMDR was between 35.7–28.7% for boys, and 42.9–29.7% for girls. Children 4–8 years old showed the highest percentages under the AMDR. The EFSA sets the Reference Intake range (RI) for carbohydrates at 45–60%En for children over 1 year of age [51], and only 0.2% of boys and 0.1% of girls age 1–3 years had usual intake that exceeded that range.

Total fat accounted for 34.6% of total energy intake. The proportion of participants with usual En% intake from total fat above the upper limit of the AMDR was between 36.5 and 47.5% for boys and 38.6 and 58.4% for girls, children and adolescents age 4–17. Usual fat intake of 54.6% of boys and 55.4% of girls aged 1–3 was below 35% En, whereas there were no children over 4-year-old with intakes lower than 20% En, i.e. the lower limits set by EFSA for these age groups [51]. %En from SFA and MUFAs was similar for boys and girls, increasing from 6–12 months to 4–8 years and decreasing after that. The usual median intake of %En from PUFAs was 5.0%, similar across ages and gender.

Sensitivity analysis showed that the exclusion of misreporters in the group of children between 6 and 12 months shifted distribution to the left whereas in adolescents it shifted it to the right. The exclusion of misreporters mainly resulted in slight differences in the percentage of population out the AMDR, lower than 3 percentage points so it does not significantly modify the results and conclusions of this study.

## Discussion

ENALIA provides the most recent data on food and beverage consumption and eating habits of children and adolescents in Spain with a large and representative sample. The ENALIA study is of great interest not only because it provides data with which to evaluate the nutritional adequacy of a representative sample of Spanish children and adolescents ranging from age 6 months to 17 years, but also because it facilitates comparison with results from other European countries that have used the same methodology. Moreover, it will be the reference study to monitor the diet of Spanish children and adolescents in the future. In general, results show that the majority of the population consumed proportions of macronutrients within the acceptable ranges, except for approximately one-third of the population that was outside of the AMDR for carbohydrates and fats.

At national level, comparisons with the last national reference study for dietary assessment in children and adolescents, the EnKid study [20], suggest that total energy intake has declined slightly (the average total energy intake in the EnKid study was 2078 Kcal/day) and that macronutrient distribution has improved slightly, the %En from carbohydrates increasing and the %En from fats decreasing over the last 20 years (EnKid study: 42.7% %En from carbohydrates and 39.6% %En from fats). A random sample of 3534 people between the ages of 2 and 24 were interviewed by 24-h recalls and a second 24-h recall was made in 30% of the sample in the EnKid study. A food-frequency questionnaire and other questions relating to lifestyle, knowledge and food preference rounded out the food consumption data. It is important to bear in mind that it is difficult to draw comparisons between our results and EnKid (specifically absolute data) and other studies on dietary intake [53–56] due to differences in dietary assessment methods, underlying food composition tables, study population, age categories chosen and statistical estimation procedures. The ultimate objective of ENALIA is to obtain dietary information to gain a better understanding of the nutritional profile in the Spanish population and to compare it to other European countries taking part in the EU Menu Project. However, until those data are available, comparisons are made with the last national and European food consumption surveys in infants, children, and adolescents where usual energy and macronutrient intakes have been estimated.

We would like to highlight results by age group since macronutrient distribution and specific recommendations vary according to age. Among infants (6–12 months), more than half of %En comes from carbohydrates, a third from fats and the smallest percentage from proteins. In young children (age 1–3), proteins account for a higher %En, with a small percentage exceeding the upper limit of the AMDR for protein intake. One-third did not reach the lower limit of the AMDR for carbohydrate intake and a small percentage were outside of the AMDR for fat intake, either below or above the limits. The latest national data on infants and young children (1–3 years) are described in the ALSAMA study [57]. Protein intake continues to be high and there is a slight improvement in carbohydrate and fat intake. In Europe, some studies have recently been conducted targeting this age group [55, 58–60]. Results were generally similar to ours, highlighting an excess of energy from proteins and SFAs to the detriment of PUFAs. The high protein intake in children below 4 years of age is a concern. In Spain, 26.1% of children are overweight and 18.63% are obese [61] and high protein intake in infancy and early childhood has been associated with increased growth and a higher body mass index (BMI) in childhood [21, 62, 63]. In the school-aged group we found that 4–8 year olds had the highest %En from carbohydrates below the reference values and the highest %En from fats over the reference values. In a study by Bornhorst et al. [64] conducted on 8611 children age 2–9, mean results are consistent with our results. Specifically, the mean %En from fat and protein for the entire sample was 32.3 and 15.7%, respectively, slightly lower for fats and proteins compared to our data and slightly higher for carbohydrates (52.1%En). As for the adolescent population, results from the HELENA study [65] targeting 12.5–17.5-year-old adolescents from eight cities in Europe in 2008 showed higher energy intake (from 2255 to 2806 Kcal/day) and a similar caloric profile to our study, the differences being in proteins and SFA intake which exceeded that of the ENALIA population. Protein intake was about twice as high as recommended and SFA was about 40% higher than recommended [66].

The energy intake and macronutrient distribution needed are in accordance with rapid development and growth at this stage where carbohydrates are essential for energy and are the main contributors of glucose for the brain and with fats facilitating the absorption of fat-soluble vitamins and supporting neurodevelopment [22].

However, diet quality is as important as quantity. Regarding fat intake, EFSA does not establish minimum or maximum intake of MUFA due to a lack of supporting scientific evidence [67]. However in Europe institutions such as EFSA, the Food and Agriculture Organization (FAO) or SENC warn that total fat consumption should not exceed 35% of total energy [67–69], provided this limitation is not at the expense of MUFAs. MUFA intake recommendations vary between 7 and 20% of total energy [68]. According to the SENC, in Spain the nutritional target for MUFA is > 20% of total energy [69]. These figures coincide with the findings in the study called Prevención con Dieta Mediterránea (Prevention with the Mediterranean Diet—PREDIMED) [70] conducted with the participation of 7,447 people and which analyzed the effect that three different diets had on the risk of cardiovascular disease (a Mediterranean diet supplemented with extra virgin olive oil, a Mediterranean diet supplemented with dried fruit and a control diet (advice to reduce fat in the diet)). Results indicated reduced risk of cardiovascular disease and mortality where MUFA accounted for 22% of total energy intake (mainly virgin olive oil). Based on these results, a target of MUFA accounting for 20–25% of total energy with olive oil as the main dietary source was proposed. A higher than recommended intake of total fat, including SFAs, could have a severe impact on health [31, 71]. Trans Fatty Acids and Saturated Fatty Acids are considered the main cardiovascular risk factors meaning that their consumption in a nutritionally balanced diet should be as low as possible [50]. More than 10% of total energy came from SFAs in all age groups of the study population, to the detriment of PUFA. This also occurs in other European countries such as Italy [56] and the United Kingdom [54] where the energy provided by SFAs varies between 11.5 and 14.8% and in France [53] where SFAs and MUFAs are the most prevalently consumed fats (47 and 38% of total fats, respectively). Nevertheless, the correlation between health and the consumption of different fatty acids is currently being questioned [72–74]. For instance, some saturated fatty acids coming from milk and dairy products have positive effects on the adult population, the most recent evidence suggesting that the consumption of dairy products contributes to meeting nutrient recommendations and may provide protection against the most prevalent chronic diseases with few adverse effects reported [75]. Moreover, in a recent study of a group of children under age 7 [76], no correlation was observed between the intake of total fat or SFA, MUFAs, or PUFAs and growth, adiposity and cardio-metabolic health. More research is needed to determine the true role of the different SFAs on health, especially in this age group and particularly those from dairy products. Regarding fibre, several studies have demonstrated that sufficient fibre intake is linked to important beneficial health effects including reduced risk of cardiovascular disease, Type II Diabetes [77], some types of cancer [78] and maintaining body weight. The average daily fibre consumption of the study population was 15.5 g/day, higher in males and increasing with age. This value is slightly above the values for France (12.6 g/day) [53] and Italy (14.5 g/day) [56].

As lifestyle plays an important role in determining long-term preferences and health behaviours, a lifestyle approach that starts early and encourages long-term changes is needed [79]. Moreover, the IDEFICS study [80] highlights the importance of families and the environment on the lifestyle and eating habits of children and adolescents. These two factors should, therefore, be borne in mind when addressing healthy food policies. Moreover, informing and empowering families on healthy eating is an area that should be targeted for development since it will have an impact on the nutritional status of children and adolescents and contribute to reducing obesogenic behaviours.

The exclusion of misreporters does not significantly modify the results of our study. Between 0.6 and 19.8% of the sample were considered underestimates, lower than in other European [54, 56] and national studies [81]. The exclusion of underestimates is a controversial topic. The exclusion of under-/over-reporters could have introduced selection bias as misreporters might have a special food choice or eating behaviour. In addition, under-reporting includes both under-recording and under-eating and some over-reporters could have eaten much more than usual during the study period as well. It has also been suggested that low energy reporting may be just as common among plausible energy reporters as among those defined as under-reporters [82] so that selectively excluding those with implausible energy intakes could bias the results. Furthermore, during childhood diet tends to be highly variable from day to day making the identification of under-reporters difficult [83]. Therefore, in line with EFSA recommendations [32], we did not exclude potential misreporters from the analysis.

The results of this study should be interpreted in the light of its limitations and strengths. Special care was taken in the design of the study to ensure that sampling was carried out very carefully and that the sample was representative of the Spanish target population. However, as in all nutritional studies, it is possible that individuals particularly concerned about their health, diet or body weight may be more likely to agree to participate. In addition, dietary assessment by means of self-referenced surveys is strongly affected by misreporting (both under- and over-reporting) giving rise to measurement error [22, 43]. Although parents may be reliable reporters of their children’s food intake at home, meals out of parental control are prone to misreporting as is the estimation of their portion sizes. Moreover, when the questionnaire is answered by the parents the results could be overestimated, specifically when it comes to portion sizes [84]. In addition, when information about socially reprehensible behaviour such as an unhealthy diet is collected, the results are often underestimated and this could also be the case with children and adolescents from age 11 to 17 [43]. On the other hand, for children under 10, meals eaten at the school may not have been collected accurately, since parents and caretakers requested information from school canteen personnel. However, the methodology used in the present study, based on two non-consecutive one-day food diaries, allows to collect food and beverages consumption from parents and different caretakers, depending on the location of the child [16, 85]. In addition, school canteen personnel know very well the children in their care and know their food preferences and aversions, and the amount of the food they usually eat of each served dish.

One of the major strengths of this study, in addition to its representativeness of the total population by age group and sex, is its large sample size of children and adolescents. Moreover, methods validated and agreed in Europe [32] were rigorously applied when collecting food consumption data through 1-day food diaries/24-h dietary recalls which are less prone to systematic bias than other food survey tools. Two dietary assessment methodologies which assessed and compared the two methods for different age groups was used as recommended by EFSA [16, 86]. While more costly, dietary records are better for collecting detailed information [87] and estimating usual intake provided they are done on non-consecutive days. This is the most appropriate way to gather information about participants under age 7 (pre-schoolers) as parents and other caregivers act as surrogate responders [88]. Once school starts it is harder for them to know details about the food if they have lunch at school. The “24-h recall” method was chosen as the best way to gather information from children over 10-year-old and adolescents since the response rate is higher [89] thus increasing representativeness. But this method entails greater memory bias. In our study, we believe this bias was reduced thanks to the support tools used to recall meals and portions, home visits and the use of ENIA-Soft.

The ISU method corrected data for day-to-day variation, although we should still bear in mind that the true intake distribution remains unknown because of the lack of objective validation data. There was no day-of-the-week effect since all days of the week were included and we assessed an entire year thus including seasonal variations in the diet. The food composition tables may not accurately reflect the nutrient composition of the specific foods consumed. Variability in the composition of foods, likely due to seasonal differences, cultivar, or variety. The Spanish Food Composition Tables used included enriched/fortified foods commonly available in Spain and additional composition data for specific brands were taken into account. Another limitation in the dietary data is that breast milk cannot be precisely measured. Finally, the reason for dietary intake not meeting dietary guidelines cannot be confirmed due to the lack of biochemical data and functional parameters.

In conclusion, monitoring of nutrient intake is essential to gain insight into the needs of the target population and to guide healthy eating policies, specifically in the case of children and adolescents. ENALIA contains recent and reliable data to determine the nutritional status of Spanish children and adolescents. The study could be repeated so as to assess trends. Additional studies would likewise inform national nutritional guidelines and the development of consensus recommendations. Our results suggest that Spanish children and adolescents can improve macronutrient distribution by reducing fat intake and increasing carbohydrate intake in all age groups and decreasing protein intake, especially in the youngest ones. This information highlights the importance of monitoring nutritional status and implementing health education programs targeting children and adolescents. It is important to reinforce nutrition and health messages aimed at parents and caregivers and to encourage healthy, scientifically evaluated lunch programs at schools. This information provided by the ENALIA survey on energy and macronutrient intake, complete with the micronutrients already described [33], provides the national reference needed to take action contributing to an improvement in the nutritional status of the population and the common goal of reducing childhood obesity.

## Electronic supplementary material

Below is the link to the electronic supplementary material.

Supplementary material 1 (DOCX 29 KB)
