# Supplementary material for: Adequacy of usual macronutrient intake and macronutrient distribution in children and adolescents in Spain: A National Dietary Survey on the Child and Adolescent Population, ENALIA 2013–2014
Source: Eur J Nutr. 2018 May 22;58(2):705–19. doi: 10.1007/s00394-018-1676-3 (PMC6437129; doi:10.1007/s00394-018-1676-3)
Supplement: Supplementary file 1 — Supplementary material 1 (DOCX 29 KB) [file 394_2018_1676_MOESM1_ESM.docx]

Suplemental Table 1.- Usual intakes (from food and beverage sources only) of macronutrients in Spanish children and adolescents and inadequate intakes excluding misreporters

|  | Boys | | | | | | | | **Girls** | | | | | | | |
| --- | --- | --- | --- | --- | --- | --- | --- | --- | --- | --- | --- | --- | --- | --- | --- | --- |
|  | **mean±DS** | **P10** | **P25** | **P50** | **P75** | **P90** | **EAR** | **<EAR** | **mean±DS** | **P10** | **P25** | **P50** | **P75** | **P90** | **EAR** | **<EAR** |
| **Protein (g)** |  |  |  |  |  |  |  |  |  |  |  |  |  |  |  |  |
| 6-12 months | 32,7±8,7 | 22,1 | 26,5 | 32,0 | 38,1 | 44,2 |  |  | 29,9±7,5 | 20,7 | 24,6 | 29,4 | 34,6 | 39,8 |  |  |
| 1-3 years | 60,9±9,7 | 48,8 | 54,1 | 60,3 | 67,1 | 73,6 | 11 | 0,0 | 56,3±10,3 | 43,7 | 49,0 | 55,6 | 62,8 | 70,0 | 11 | 0,0 |
| 4-8 years | 77,9±10,7 | 64,5 | 70,4 | 77,5 | 84,9 | 91,8 | 19 | 0,0 | 70,0±6,1 | 62,4 | 65,7 | 69,7 | 74,0 | 78,0 | 19 | 0,0 |
| 9-13 years | 90,6±8,8 | 79,6 | 84,5 | 90,3 | 96,3 | 102,1 | 34 | 0,0 | 79,7±9,8 | 67,4 | 72,9 | 79,4 | 86,1 | 92,4 | 34 | 0,0 |
| 14-17 years | 109,9±13,5 | 93,1 | 100,6 | 109,3 | 118,6 | 127,5 | 52 | 0,0 | 85,3±12,7 | 69,4 | 76,5 | 84,8 | 93,6 | 101,8 | 46 | 0,0 |
| **Carbohydrates (g)** |  |  |  |  |  |  |  |  |  |  |  |  |  |  |  |  |
| 6-12 months | 140,6±19,0 | 116 | 128 | 141 | 153 | 165 |  |  | 125,1±20,7 | 99 | 111 | 125 | 139 | 152 |  |  |
| 1-3 years | 171,1±23,8 | 141 | 154 | 170 | 187 | 202 | 100 | 0,0 | 153,9±19,1 | 129 | 141 | 154 | 167 | 178 | 100 | 0,0 |
| 4-8 years | 214,1±29,6 | 177 | 193 | 213 | 233 | 253 | 100 | 0,0 | 186,9±24,6 | 156 | 170 | 186 | 203 | 219 | 100 | 0,0 |
| 9-13 years | 251,5±28,2 | 216 | 232 | 251 | 270 | 288 | 100 | 0,0 | 221,1±25,6 | 189 | 203 | 220 | 238 | 255 | 100 | 0,0 |
| 14-17 years | 293,9±46,1 | 236 | 262 | 292 | 324 | 354 | 100 | 0,0 | 224,2±31,5 | 185 | 202 | 223 | 244 | 265 | 100 | 0,0 |
| **Fiber (g)** |  |  |  |  |  |  |  |  |  |  |  |  |  |  |  |  |
| 6-12 months | 9,2±3,0 | 5,4 | 7,1 | 9,1 | 11,2 | 13,2 |  |  | 8,2±2,7 | 5,1 | 6,4 | 7,9 | 9,7 | 11,7 |  |  |
| 1-3 years | 12,5±3,4 | 8,5 | 10,1 | 12,1 | 14,5 | 17,0 |  |  | 11,4±3,7 | 7,2 | 8,8 | 10,9 | 13,5 | 16,3 |  |  |
| 4-8 years | 15,9±2,4 | 12,9 | 14,1 | 15,7 | 17,4 | 19,1 |  |  | 15,3±3,3 | 11,4 | 13,0 | 15,0 | 17,2 | 19,6 |  |  |
| 9-13 years | 18,7±3,8 | 14,2 | 16,0 | 18,4 | 21,0 | 23,7 |  |  | 17,7±4,5 | 12,3 | 14,4 | 17,2 | 20,4 | 23,7 |  |  |
| 14-17 years | 22,5±5,2 | 16,3 | 18,8 | 22,0 | 25,6 | 29,4 |  |  | 19,1±4,5 | 13,8 | 15,9 | 18,6 | 21,7 | 25,1 |  |  |
| **Lipids (g)** |  |  |  |  |  |  |  |  |  |  |  |  |  |  |  |  |
| 6-12 months | 39,7±7,8 | 30,2 | 34,2 | 39,1 | 44,5 | 50,0 |  |  | 36,1±4,4 | 30,6 | 33,1 | 36,0 | 39,0 | 41,9 |  |  |
| 1-3 years | 56,9±10,9 | 43,3 | 49,2 | 56,3 | 63,9 | 71,3 |  |  | 51,1±8,2 | 41,1 | 45,3 | 50,6 | 56,3 | 61,9 |  |  |
| 4-8 years | 71,5±6,4 | 63,5 | 67,1 | 71,3 | 75,7 | 79,8 |  |  | 65,2±4,8 | 59,1 | 61,9 | 65,1 | 68,4 | 71,4 |  |  |
| 9-13 years | 83,8±11,4 | 69,6 | 75,9 | 83,3 | 91,1 | 98,6 |  |  | 74,0±11,3 | 60,1 | 66,1 | 73,4 | 81,2 | 88,8 |  |  |
| 14-17 years | 95,6±15,3 | 76,8 | 84,8 | 94,6 | 105,3 | 115,7 |  |  | 74,5±15,6 | 54,4 | 63,5 | 74,3 | 85,4 | 95,0 |  |  |
| **SFA (g)** |  |  |  |  |  |  |  |  |  |  |  |  |  |  |  |  |
| 6-12 months | 9,6±4,8 | 4,6 | 6,2 | 8,5 | 12 | 16,3 |  |  | 9,1±5,0 | 3,4 | 5,4 | 8,2 | 11,8 | 15,7 |  |  |
| 1-3 years | 19.8±7,0 | 10,8 | 14,6 | 19,5 | 24,6 | 29,2 |  |  | 18,3±6,5 | 10,3 | 13,6 | 17,8 | 22,4 | 26,9 |  |  |
| 4-8 years | 27,1±4,6 | 21,4 | 23,9 | 26,8 | 30 | 33,1 |  |  | 24,0±4,0 | 19,1 | 21,2 | 23,7 | 26,5 | 29,2 |  |  |
| 9-13 years | 29,4±5,6 | 22,5 | 25,4 | 29 | 32,9 | 36,7 |  |  | 26,7±5,4 | 20,1 | 22,9 | 26,3 | 30,1 | 33,7 |  |  |
| 14-17 years | 32,0±7,1 | 23,2 | 26,9 | 31,5 | 36,5 | 41,4 |  |  | 24,1±5,1 | 17,8 | 20,5 | 23,8 | 27,4 | 30,9 |  |  |
| **MUFA (g)** |  |  |  |  |  |  |  |  |  |  |  |  |  |  |  |  |
| 6-12 months | 11,8±4,6 | 6,3 | 8,5 | 11,3 | 14,6 | 17,9 |  |  | 11,3±4,0 | 6,4 | 8,4 | 10,9 | 13,8 | 16,6 |  |  |
| 1-3 years | 19,2±6,1 | 11,6 | 14,9 | 18,8 | 23,1 | 27,3 |  |  | 17,8±5,0 | 11,7 | 14,3 | 17,5 | 20,9 | 24,3 |  |  |
| 4-8 years | 27,4±4,3 | 22,1 | 24,4 | 27,1 | 30,1 | 33 |  |  | 25,9±2,6 | 22,6 | 24,1 | 25,8 | 27,6 | 29,3 |  |  |
| 9-13 years | 32,5±5,5 | 25,7 | 28,7 | 32,2 | 36 | 39,6 |  |  | 28,0±5,1 | 21,6 | 24,3 | 27,6 | 31,2 | 34,7 |  |  |
| 14-17 years | 35,3±7,5 | 26,2 | 30 | 34,7 | 40 | 45,2 |  |  | 29,1±7,0 | 20,3 | 24,2 | 28,9 | 33,7 | 38,3 |  |  |
| **PUFA (g)** |  |  |  |  |  |  |  |  |  |  |  |  |  |  |  |  |
| 6-12 months | 6,0±1,1 | 4,8 | 5,3 | 6 | 6,7 | 7,4 |  |  | 5,8±0,9 | 4,8 | 5,2 | 5,7 | 6,3 | 7 |  |  |
| 1-3 years | 8,4±2,2 | 5,9 | 6,9 | 8,1 | 9,6 | 11,3 |  |  | 7,7±1,3 | 6,2 | 6,8 | 7,5 | 8,4 | 9,3 |  |  |
| 4-8 years | 10,5±0,8 | 9,6 | 10 | 10,5 | 11 | 11,5 |  |  | 9,1±1,7 | 7,1 | 7,9 | 8,9 | 10,1 | 11,3 |  |  |
| 9-13 years | 12,2±2,6 | 9,1 | 10,4 | 12 | 13,8 | 15,6 |  |  | 11,0±2,3 | 8,2 | 9,3 | 10,7 | 12,4 | 14 |  |  |
| 14-17 years | 13,6±2,9 | 10,1 | 11,5 | 13,3 | 15,3 | 17,4 |  |  | 11,4±2,5 | 8,4 | 9,7 | 11,2 | 12,9 | 14,7 |  |  |
| **Cholesterol (mg)** |  |  |  |  |  |  |  |  |  |  |  |  |  |  |  |  |
| 6-12 months | 122±68 | 46 | 71 | 109 | 163 | 219 |  |  | 113±67 | 38 | 64 | 101 | 150 | 203 |  |  |
| 1-3 years | 229±88 | 126 | 165 | 218 | 281 | 347 |  |  | 220±93 | 110 | 151 | 207 | 276 | 347 |  |  |
| 4-8 years | 328±55 | 261 | 290 | 324 | 363 | 400 |  |  | 296±41 | 246 | 267 | 294 | 322 | 349 |  |  |
| 9-13 years | 366±54 | 298 | 327 | 362 | 400 | 437 |  |  | 294±79 | 199 | 238 | 287 | 342 | 398 |  |  |
| 14-17 years | 401±73 | 312 | 350 | 397 | 447 | 497 |  |  | 331±69 | 249 | 282 | 324 | 373 | 422 |  |  |

**Suplemental table 2.** Distribution of relative usual intakes (from food and beverage sources only) of macronutrients (percent of the total energy intake, %En) and PUFA+MUFA/SFA ratio in Spanish children and adolescents and inadequate intakes excluding misreporters

|  | Boys | | | | | | | | | **Girls** | | | | | | | | |  |
| --- | --- | --- | --- | --- | --- | --- | --- | --- | --- | --- | --- | --- | --- | --- | --- | --- | --- | --- | --- |
|  | **mean±DS** | **P10** | **P25** | **P50** | **P75** | **P90** | **AR** | **<AR** | **>AR** | **mean±DS** | **P10** | **P25** | **P50** | **P75** | **P90** | **AR** | **<AR** | **>AR** | |
| **Protein (% kcal)** |  |  |  |  |  |  |  |  |  |  |  |  |  |  |  |  |  |  | |
| 6-12 months | 12,1±1,9 | 9,8 | 10,7 | 11,9 | 13,2 | 14,5 |  |  |  | 12,2±1,7 | 10,1 | 11,0 | 12,1 | 13,3 | 14,5 |  |  |  | |
| 1-3 years | 16,6±1,9 | 14,3 | 15,3 | 16,5 | 17,8 | 19,1 | 5-20 | 0,0 | 4,5 | 17,0±2,2 | 14,3 | 15,5 | 16,9 | 18,4 | 19,9 | 5-20 | 0,0 | 9,4 | |
| 4-8 years | 17,0±1,4 | 15,3 | 16,1 | 17,0 | 17,9 | 18,9 | 10-30 | 0,0 | 0,0 | 17,2±1,5 | 15,3 | 16,1 | 17,1 | 18,1 | 19,2 | 10-30 | 0,0 | 0,0 | |
| 9-13 years | 16,9±1,5 | 15,1 | 15,9 | 16,8 | 17,9 | 18,9 | 10-30 | 0,0 | 0,0 | 16,8±1,9 | 14,5 | 15,5 | 16,8 | 18,1 | 19,3 | 10-30 | 0,0 | 0,0 | |
| 14-17 years | 17,5±1,6 | 15,5 | 16,4 | 17,4 | 18,5 | 19,6 | 10-30 | 0,0 | 0,0 | 17,7±2,2 | 14,9 | 16,1 | 17,6 | 19,1 | 20,6 | 10-30 | 0,0 | 0,0 | |
| **Carbohydrates (% kcal)** |  |  |  |  |  |  |  |  |  |  |  |  |  |  |  |  |  |  | |
| 6-12 months | 52,8±4,3 | 47,1 | 50,0 | 53,0 | 55,8 | 58,0 |  |  |  | 52,2±4,2 | 46,9 | 49,4 | 52,2 | 55,0 | 57,6 |  |  |  | |
| 1-3 years | 47,3±4,4 | 41,7 | 44,3 | 47,3 | 50,2 | 52,9 | 45-65 | 30,3 | 0,0 | 46,5±4,3 | 41,0 | 43,6 | 46,5 | 49,4 | 52,0 | 45-65 | 36,0 | 0,0 | |
| 4-8 years | 46,5±3,5 | 42,0 | 44,1 | 46,5 | 48,9 | 51,0 | 45-65 | 33,9 | 0,0 | 45,3±2,9 | 41,7 | 43,4 | 45,3 | 47,2 | 49,0 | 45-65 | 45,5 | 0,0 | |
| 9-13 years | 46,6±2,8 | 43,0 | 44,8 | 46,6 | 48,5 | 50,2 | 45-65 | 27,9 | 0,0 | 46,6±3,2 | 42,6 | 44,5 | 46,6 | 48,8 | 50,7 | 45-65 | 30,6 | 0,0 | |
| 14-17 years | 46,9±3,4 | 42,5 | 44,7 | 47,0 | 49,2 | 51,1 | 45-65 | 27,9 | 0,0 | 46,3±3,8 | 41,4 | 43,7 | 46,3 | 48,9 | 51,3 | 45-65 | 37,1 | 0,0 | |
| **Fats (% kcal)** |  |  |  |  |  |  |  |  |  |  |  |  |  |  |  |  |  |  | |
| 6-12 months | 33,6±5,2 | 27,2 | 30,0 | 33,3 | 36,9 | 40,4 |  |  |  | 34,1±4,2 | 28,7 | 31,3 | 34,1 | 37,0 | 39,6 |  |  |  | |
| 1-3 years | 34,5±3,3 | 30,3 | 32,3 | 34,6 | 36,8 | 38,7 | 30-40 | 8,6 | 4,6 | 34,8±3,1 | 30,9 | 32,7 | 34,8 | 36,8 | 38,7 | 30-40 | 5,9 | 4,4 | |
| 4-8 years | 34,8±2,5 | 31,6 | 33,1 | 34,8 | 36,5 | 38,0 | 25-35 | 0,0 | 46,7 | 35,7±2,9 | 32,0 | 33,8 | 35,7 | 37,6 | 39,4 | 25-35 | 0,0 | 59,7 | |
| 9-13 years | 34,7±3,1 | 30,8 | 32,7 | 34,8 | 36,8 | 38,6 | 25-35 | 0,1 | 47,3 | 34,7±2,8 | 31,1 | 32,8 | 34,7 | 36,6 | 38,3 | 25-35 | 0,0 | 45,9 | |
| 14-17 years | 33,9±3,6 | 29,3 | 31,4 | 33,8 | 36,2 | 38,5 | 25-35 | 0,5 | 36,9 | 34,0±3,9 | 29,0 | 31,4 | 34,0 | 36,7 | 39,1 | 25-35 | 1,0 | 40,1 | |
| **SFA (% kcal)** |  |  |  |  |  |  |  |  |  |  |  |  |  |  |  |  |  |  | |
| 6-12 months | 8,2±4,8 | 3,4 | 4,7 | 6,9 | 10,6 | 15,2 |  |  |  | 8,4±5,0 | 2,8 | 4,5 | 7,1 | 11,0 | 14,6 |  |  |  | |
| 1-3 years | 11,8±3,3 | 7,4 | 9,5 | 12,0 | 14,2 | 16,0 |  |  |  | 12,0±3,7 | 7,1 | 9,4 | 12,1 | 14,4 | 16,4 |  |  |  | |
| 4-8 years | 13,1±1,3 | 11,4 | 0,0 | 13,0 | 13,9 | 14,6 |  |  |  | 13,1±1,8 | 10,8 | 11,9 | 13,1 | 14,2 | 15,3 |  |  |  | |
| 9-13 years | 12,6±1,4 | 10,8 | 0,0 | 12,5 | 13,4 | 14,2 |  |  |  | 12,8±1,5 | 10,9 | 11,8 | 12,8 | 13,7 | 14,6 |  |  |  | |
| 14-17 years | 12,0±1,5 | 10,2 | 0,0 | 11,9 | 12,9 | 13,7 |  |  |  | 11,4±1,2 | 9,8 | 10,5 | 11,3 | 12,1 | 12,8 |  |  |  | |
| **MUFA (% kcal)** |  |  |  |  |  |  |  |  |  |  |  |  |  |  |  |  |  |  | |
| 6-12 months | 9,8±3,9 | 5,0 | 6,9 | 9,4 | 12,2 | 15,0 |  |  |  | 9,7±4,0 | 4,5 | 6,7 | 9,5 | 12,5 | 15,1 |  |  |  | |
| 1-3 years | 11,2±2,7 | 7,7 | 9,3 | 11,2 | 13,0 | 14,8 |  |  |  | 11,6±2,6 | 8,4 | 9,9 | 11,6 | 13,4 | 15,0 |  |  |  | |
| 4-8 years | 13,2±1,7 | 11,0 | 12,0 | 13,2 | 14,4 | 15,5 |  |  |  | 14,2±1,5 | 12,3 | 13,2 | 14,2 | 15,2 | 16,2 |  |  |  | |
| 9-13 years | 13,8±1,8 | 11,5 | 12,5 | 13,7 | 15,0 | 16,1 |  |  |  | 13,3±1,3 | 11,7 | 12,4 | 13,3 | 14,2 | 15,0 |  |  |  | |
| 14-17 years | 13,2±2,1 | 10,7 | 11,8 | 13,1 | 14,6 | 16,0 |  |  |  | 13,8±2,2 | 11,0 | 12,4 | 13,8 | 15,3 | 16,6 |  |  |  | |
| **PUFA (% kcal)** |  |  |  |  |  |  |  |  |  |  |  |  |  |  |  |  |  |  | |
| 6-12 months | 4,9±0,5 | 4,3 | 4,6 | 4,9 | 5,2 | 5,6 |  |  |  | 4,9±0,4 | 4,5 | 4,7 | 4,9 | 5,2 | 5,4 |  |  |  | |
| 1-3 years | 4,9±0,8 | 4,0 | 4,4 | 4,9 | 5,4 | 6,0 |  |  |  | 5,0±0,6 | 4,2 | 4,5 | 4,9 | 5,3 | 5,8 |  |  |  | |
| 4-8 years | 5,1±0,1 | 5,0 | 5,1 | 5,1 | 5,2 | 5,3 |  |  |  | 4,8±0,7 | 4,0 | 4,3 | 4,8 | 5,3 | 5,8 |  |  |  | |
| 9-13 years | 5,1±0,7 | 4,3 | 4,6 | 5,1 | 5,5 | 6,0 |  |  |  | 5,2±1,0 | 4,0 | 4,5 | 5,1 | 5,8 | 6,5 |  |  |  | |
| 14-17 years | 5,2±0,6 | 4,4 | 4,7 | 5,1 | 5,5 | 5,9 |  |  |  | 5,3±0,7 | 4,6 | 4,9 | 5,3 | 5,8 | 6,2 |  |  |  | |
